# Integrating VYC‐12L With Energy‐Based Devices for Skin‐Quality Improvement: Global Expert Considerations for Safe and Effective Outcomes

**DOI:** 10.1111/jocd.70352

**Published:** 2025-07-24

**Authors:** Shannon Humphrey, Sylvia Ramirez, Ileana E. Arreola Jáuregui, Wonseok Choi, Krishan M. Kapoor, Mansi Mukherjee, Fabiana Wanick, Reha Yavuzer, Smita Chawla, Carola de la Guardia

**Affiliations:** ^1^ Humphrey Cosmetic Dermatology Vancouver British Columbia Canada; ^2^ Department of Dermatology and Skin Science University of British Columbia Vancouver British Columbia Canada; ^3^ Cutis Medical Laser Clinics Singapore; ^4^ DERMACenter Clínica Dermatológica Guadalajara Mexico; ^5^ V Plastic Surgery Daegu Korea; ^6^ Anticlock Clinic Chandigarh India; ^7^ Kaya Skin Clinic Dubai UAE; ^8^ Fabiana Wanick Clínica de Dermatologia Rio de Janeiro Brazil; ^9^ VKV American Hospital and Rene Clinic Istanbul Turkey; ^10^ Clinical Development Allergan Aesthetics, an AbbVie Company Irvine California USA; ^11^ Global Aesthetics Medical Affairs Allergan Aesthetics, an AbbVie Company Madrid Spain

**Keywords:** energy‐based device, hyaluronic acid filler, skin quality, VYC‐12L

## Abstract

**Background:**

Energy‐based devices (EBDs) and injectable hyaluronic acid gels, such as VYC‐12L, are each effective treatment modalities for improving skin quality.

**Objectives:**

To assess the rationale and potential benefits of adding VYC‐12L to EBD treatments, and consider how they can be practically integrated into a single treatment plan.

**Methods:**

Eight clinicians with various specialties and extensive experience of these modalities completed a written questionnaire and were individually interviewed. Their collective clinical expertise and experience form the basis of this guidance.

**Results:**

Using both VYC‐12L and EBDs within a single treatment plan offers many potential benefits for improving skin quality—based on mechanistic synergies and complementary abilities to address different attributes and multiple anatomical layers. Careful consideration should be given to appropriate sequencing. VYC‐12L and non‐ablative EBDs can be used on the same day, but treatment fields must be appropriately managed and aseptic technique rigorously upheld. When administering VYC‐12L, maintaining the correct injection depth is essential to positive outcomes. Practitioners should undertake appropriate training and select the injection tools that optimize control.

**Conclusions:**

Multimodal treatment using VYC‐12L and EBDs together can provide a more comprehensive, tailored approach to skin‐quality improvement, delivering high levels of patient satisfaction.

## 1 Introduction

Improvement of skin quality is an increasingly common treatment goal in aesthetic medicine, and can have a significant impact on self‐perception and quality of life [1, 2, 3]. However, until recently, ‘skin quality’ was an imprecise concept that lacked proper clinical classification. This is now changing through the definition of various contributory attributes—including those that are purely visible (e.g., dullness, redness) and those that may be considered as mechanical (e.g., elasticity, firmness) and/or topographical (e.g., fine lines, dryness) [3]. Although such frameworks cannot necessarily cover all of the many different desires that patients have for their skin, they are very useful for facilitating more consistent and objective analyses.

A variety of methods can be used to address these attributes, including energy‐based devices (EBDs), injectable products (such as hyaluronic acid [HA] gels), chemical peels, microneedling techniques, and topical agents.

With regard to EBDs, many technologies are available. These include ablative and non‐ablative lasers, intense pulsed light (IPL), radiofrequency (RF; with or without microneedling), and ultrasound (high‐intensity focused [HIFU] or microfocused [MFU]) [4, 5, 6]. They each use different wavelengths of electromagnetic or sound energy, but all work primarily through the controlled creation of micro‐injuries that then trigger the normal healing process and enhanced production of collagen and elastin. According to the aims of treatment, the energy can be specifically directed at different anatomical planes, depending on the device and wavelengths used. Typically, lasers and IPL are targeted at epidermal and superficial dermal layers, RF penetrates into deeper dermal layers, and ultrasound technologies are used to target the deep dermis and underlying tissues [7, 8, 9].

Among approved HA injectables, VYC‐12L (Juvéderm VOLITE/SKINVIVE, Allergan Aesthetics, an AbbVie company, Pringy, Annecy, France) has demonstrated significant skin‐quality benefits. VYC‐12L contains 12 mg/mL of high‐molecular‐weight HA (based on a patented mix of long [> 500 kDa] and longer [> 1 MDa] HA chains) and 0.3% w/w lidocaine. It has the lowest G' in the Vycross family of products [10]. The HA in VYC‐12L is crosslinked for improved duration and has low cohesivity to facilitate spread. The extrusion profile was developed specifically for controlled injection, and efficient distribution can be achieved using a microdroplet technique. VYC‐12L is indicated for the correction of superficial cutaneous depressions, such as fine lines, and for the additional improvement of skin‐quality attributes like hydration and elasticity [11]. Safety and effectiveness were demonstrated in two large, prospective clinical trials based on deep intradermal microdroplet deposition with a 32G needle [12, 13, 14, 15]. In the first (*n* = 131), injections in the face and neck were associated with decreased skin roughness, reduction of fine lines, and improved hydration lasting up to 9 months following a single treatment; most patients returned to normal activities within 1 day post‐injection, and overall satisfaction was high (including assessments of skin radiance as an individual concept) [12, 13]. In the second trial (*n* = 202), VYC‐12L treatment of the cheeks improved skin smoothness, fine lines, and hydration through to re‐treatment at 6 months—as well as increasing patient satisfaction with their skin [14, 15]. Data from real‐world analyses have further confirmed the safety and effectiveness of VYC‐12L [16, 17, 18, 19].

Different treatment modalities can have complementary mechanisms of action, and varying effects on different anatomical layers and skin‐quality attributes. Hence, there is potential for significant synergy when using both VYC‐12L and one or more EBDs in the same treatment plan. This has not yet been assessed in prospective trials, although a large retrospective analysis of 736 patients treated with VYC‐12L plus other modalities—of whom almost half received EBD treatment—found no unique adverse events (AEs) attributable to concomitant usage [20]. Other smaller studies have also suggested that VYC‐12L can be safely and effectively used alongside EBDs, including laser and HIFU [21, 22].

The objectives of the current paper are: (1) to discuss the rationale and benefits of adding VYC‐12L to skin‐quality treatments performed with EBDs; (2) to assess how VYC‐12L and EBDs can be integrated in a single treatment plan; and (3) to share expert clinical experience and insights on optimal usage and patient management. This guidance is derived from a global group of clinicians with extensive experience of using VYC‐12L and EBDs in the same treatment plan.

## 2 Methods

In July and August 2024, all eight authors who are current aesthetic practitioners (i.e., excluding the final two authors) independently completed a written questionnaire on their clinical experiences with VYC‐12L and EBDs. This questionnaire covered aspects of their clinical practice in skin‐quality treatments, decision‐making processes, experience of utilizing both modalities in a single treatment plan, and patient management strategies. Each practitioner was then separately interviewed via video call to clarify and elaborate on their written responses. The present paper collates the joint experience of these experts, representing diverse specialties (such as dermatology, plastic surgery, and aesthetic medicine) across eight different countries around the world.

At the time of completing the questionnaire, all injectors had been using VYC‐12L in their clinical practice for 3–8 years, depending on the date of approval in their respective countries. Collectively, they have treated more than 5000 patients with VYC‐12L and injected around 20 000 syringes, putting them among the most experienced users globally. Furthermore, all of the practitioners employ at least three different types of EBD in their daily practice (Table 1).

**TABLE 1:** Use of energy‐based devices.

Author^a^	EBD modality			
	Laser	Intense pulsed light	Radiofrequency^b^	Ultrasound (HIFU/MFU)
IEAJ	✓	✓	✓	✓
WC	✓	×	✓	✓
SH	✓	✓	✓	✓
KMK	✓	×	✓	✓
MM	✓	✓	✓	✓
SR	✓	✓	✓	✓
FW	✓	✓	×	✓
RY	×	✓	✓	✓

	Laser	Intense pulsed light	Radiofrequency^b^	Ultrasound (HIFU/MFU)
Key skin‐quality attributes that can be addressed^c^	Pores Dyschromia Thin skin Crepey skin Fine lines Dullness Hyperpigmentation	Skin redness Dyschromia Hyperpigmentation Dullness	Firmness Elasticity Laxity Thin skin Crepey skin Pores	Elasticity Laxity Thin skin Crepey skin Pores

Abbreviations: EBD, energy‐based device; HIFU, high‐intensity focused ultrasound; MFU, microfocused ultrasound.

^a^ Listed in alphabetical order by surname.

^b^ May be used with or without microneedling.

^c^ The skin‐quality attributes listed are those that gained the most votes among the injectors in the author group based on a standardized list [3].

## 3 Skin‐Quality Attributes

The eight injectors were provided with a standardized list of skin‐quality attributes [3], and asked to select the top three attributes that their patients consider most important, as well as those that can be addressed with VYC‐12L or EBDs.

For their patients, the most frequently cited attributes were dullness (*n* = 4), fine lines (*n* = 3), laxity (*n* = 3), and roughness (*n* = 3) (Table 2). However, priorities will depend considerably on individual patient characteristics, such as age, sex, ethnicity, skin type, and the local climate.

**TABLE 2:** The most important skin‐quality attributes that patients want to address.

Skin‐quality attribute	Total votes
Dullness	4
Fine lines	3
Laxity	3
Roughness	3
Crepey skin	2
Dryness	2
Elasticity	2
Firmness	2
Pores	2
Dyschromia	1

*Note:* The eight injectors were provided with a standardized list of skin‐quality attributes [3], and asked to select the top three attributes that their patients consider most important. This table collates the total number of injectors voting for each attribute when considering their own patients.

**FIGURE 1:**
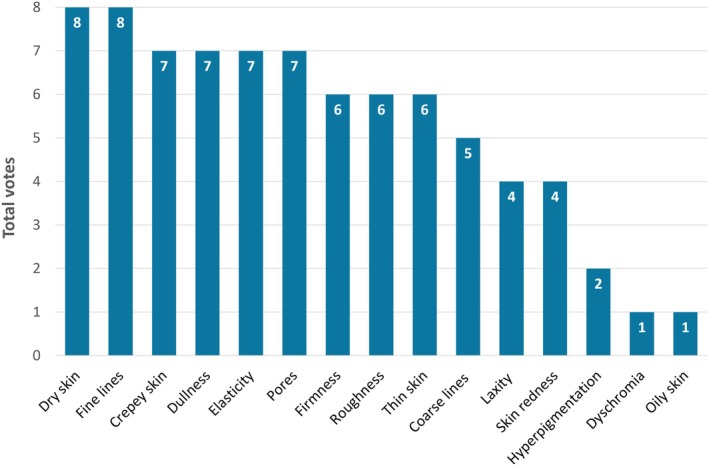
Skin‐quality attributes that can be improved with VYC‐12L. The figure shows the total votes for each attribute among the eight injectors in the author group.

The practitioners unanimously agreed that VYC‐12L effectively improves dry skin and fine lines, as per the product indication (Figure 1) [11]. Most (*n* ≥ 6) also stated that VYC‐12L can have a meaningful impact on a range of other attributes, including crepiness, dullness, elasticity, pores, firmness, roughness, and thin skin—which aligns well with patients' priorities. Many of these attributes can also be improved using EBDs (Table 1). However, dry skin and dullness are typically best addressed using VYC‐12L, particularly in patients specifically seeking enhanced hydration or improvements in “radiance”, “glow” or “luminosity”.

Importantly, the injectors in the author group use VYC‐12L in appropriate patients regardless of age, ethnicity, phototype, or damage severity, and in a wide variety of facial areas—including the cheek, forehead, temple, perioral, and periocular areas, as per clinical study data [12, 16]. In addition, many of the present authors inject VYC‐12L outside the face, for example in the neck, décolletage, hands, elbows, and knees. Some also use it in the treatment of photoaging or acne scars (in combination with subcision in more severe cases). VYC‐12L can potentially be combined with relevant EBDs for almost all such treatment indications, according to patient needs and practitioner preferences.

EBDs are often used for skin‐quality attributes or conditions that are less effectively treated with VYC‐12L. This will vary according to the specific device and protocol used, and Table 1 provides an indication of the attributes that the author group most commonly treats using different types of EBD. For example, the targeting of superficial layers with laser or IPL can be successfully used to treat skin color issues like dyschromia, redness, and hyperpigmentation, whereas the targeting of deeper tissues with ultrasound devices may be used to address laxity. RF devices are often deployed to improve skin firmness and elasticity.

Overall, multimodal approaches based on using appropriate EBD(s) alongside VYC‐12L can provide a more synergistic, comprehensive, and holistic treatment plan.

## 4 Treatment Decision‐Making With VYC‐12L and EBDs

Most of the practitioners in the current author group use VYC‐12L and EBDs in the same treatment plan with at least 30% of patients treated for skin‐quality improvement (Table 3). Both modalities can contribute significantly to the prevention and management of signs of aging skin. From a practical perspective, clinicians should at least consider using them in the same treatment plan for all eligible patients.

**TABLE 3:** Approximate proportions of patients treated with VYC‐12L and/or EBD for skin‐quality improvement who receive each modality.

Author^a^	Proportion of patients^b^		
	VYC‐12L only	EBD only	Both VYC‐12L and EBD
IEAJ	30%	30%	40%
WC	5%	85%	10%^c^
SH	20%	40%	40%
KMK	30%	40%	30%
MM	40%	30%	30%
SR	10%	60%	30%
FW	50%	20%	30%
RY	30%	30%	40%

Abbreviation: EBD, energy‐based device.

^a^ Listed in alphabetical order by surname.

^b^ Proportion receiving each modality in the same session or as part of the same treatment plan.

^c^ Most treatments at this center are performed using EBDs (as EBDs are the usual patient ‘entry point’ for skin‐quality improvements) but VYC‐12L can provide important additional benefits.

Indeed, in the clinical experience of the injectors in the current author group, there are several potential benefits of this approach (Table 4). The first is their synergistic mechanisms of action, which could make VYC‐12L a particularly valuable treatment when added to an EBD‐based protocol. For example, the two modalities have contrasting effects on hydration levels. An inevitable risk of subjecting the skin and underlying tissues to external energy using an EBD is that the heat produced may increase transepithelial water loss (TEWL) [23, 24]. Co‐treatment with VYC‐12L could play a significant role in counterbalancing this effect—owing to the hygroscopic effects of HA and increased expression of aquaporin‐3 (AQP3), a key epidermal membrane protein involved in the transport of water and glycerol [25]. Clinical trials of single treatments with VYC‐12L, based on intradermal microdroplet injections, demonstrated significant improvements in skin hydration at depths of 0.5 mm and 1.5 mm [12, 14]. Moreover, in both an ex vivo evaluation of human skin explants and an in vivo analysis of healthy subjects, single VYC‐12L treatments were associated with progressive increases in AQP3 expression in the skin and improvements in hydration of the stratum corneum [26, 27].

**TABLE 4:** Usage of VYC‐12L and EBDs together or separately: Clinical experience.

Rationale for using VYC‐12L and EBDs together	Possible reasons for using VYC‐12L only	Possible reasons for using EBDs only
Putative mechanistic synergies (e.g., contrasting effects on skin hydration) Ability to address different skin‐quality attributes Capacity to impact on multiple anatomical layers Varying speed of onset of results (may be more rapid with VYC‐12L and more gradual with EBDs) Potential for greater patient satisfaction and improved patient retention	Primary treatment goal that is particularly well‐suited (e.g., improvement of skin dryness, dullness, fine lines) Patient with budget constraints Patient with high skin phototype, concerned about potential side effects of EBD Patient with limited availability^a^ or not wanting a multisession treatment plan Patient focused on rapid results Patient averse to the idea of controlled damage and healing with EBD treatment	Skin condition or primary treatment goal that is particularly well‐suited (e.g., improvement of laxity, rosacea, melasma) Patient with budget constraints Needle‐phobic or needle‐averse patient Contraindication for VYC‐12L^b^ Patient with a high risk of significant bruising or not willing to accept such a risk

Abbreviation: EBD, energy‐based device.

^a^ For example, an individual who has traveled to the clinic from overseas.

^b^ Such as untreated epilepsy, tendency to develop hypertrophic scarring, or known hypersensitivity to hyaluronic acid or lidocaine [11].

A second possible synergy relates to the biological consequences of EBD treatments. Depending on the target anatomical layer, they typically create controlled micro‐injuries in the skin or just below, which then lead to healing and enhanced production of collagen and elastin. HA plays an important role in this process, helping to create an optimal repair milieu. For example, using in vitro models of human skin and wound healing, the injection of HA led to enhanced epidermal thickness, upregulation of collagens, increased expression of key regulators of regeneration and tissue remodeling, and downregulation of proinflammatory cytokines [28, 29]. Similarly, in an ex vivo assessment of living human skin explants, single VYC‐12L treatments increased collagen density and were associated with greater expression of another key dermal protein, fibrillin‐1 [26]. Furthermore, in a prospective study of healthy humans, genomic analysis following single intradermal VYC‐12L injections showed upregulation of a range of genes acting in the dermis and subdermal adipose tissue—involved in keratinocyte renewal, adipocyte differentiation, maintenance of the extracellular matrix, and stem‐cell regulation [27]. Injected HA may also function as a scaffold for the ingrowth of cells, including fibroblasts, and indeed, HA is a known regulator of the fibroblast response to injury [30].

Taken together, these effects suggest that skin‐quality improvements following VYC‐12L injection could be at least partly related to changes resulting from physical interaction between the product and the surrounding tissue—supporting the long‐term patient satisfaction observed in clinical trials [27].

Alongside putative mechanistic synergies, there are other compelling reasons for using VYC‐12L and EBDs in the same treatment plan (Table 4). First, they facilitate improvements in a range of different skin‐quality attributes, as noted previously, thereby yielding more holistic and comprehensive outcomes. Of course, different EBDs have varying effects depending on the device, protocol, and specific wavelengths used, and practitioners should give due consideration not only to individual needs but also the wider demographic and sociocultural background of their patients. Second, aging occurs across various anatomical planes, and no single treatment modality can address all of these. Depending on the EBD, there is potential to impact multiple layers when used alongside VYC‐12L; for example, while VYC‐12L is typically injected into the dermis, MFU can be targeted at deeper layers such as the superficial muscular aponeurotic system (SMAS), while IPL and lasers may be focused on the superficial dermis and epidermis. Third, the onset of effects is often distinct, with a more rapid impact typically evident following VYC‐12L injection, whereas EBDs usually (although not always) require a longer timeframe. All of the above feeds into the final advantage of pairing VYC‐12L and EBD—the potential for greater patient satisfaction. Indeed, a recent large multinational analysis found that multimodal treatment with EBD, HA filler, and neuromodulator on the same day led to significantly improved patient retention (a surrogate for satisfaction) compared with sequential use [31].

**FIGURE 2:**
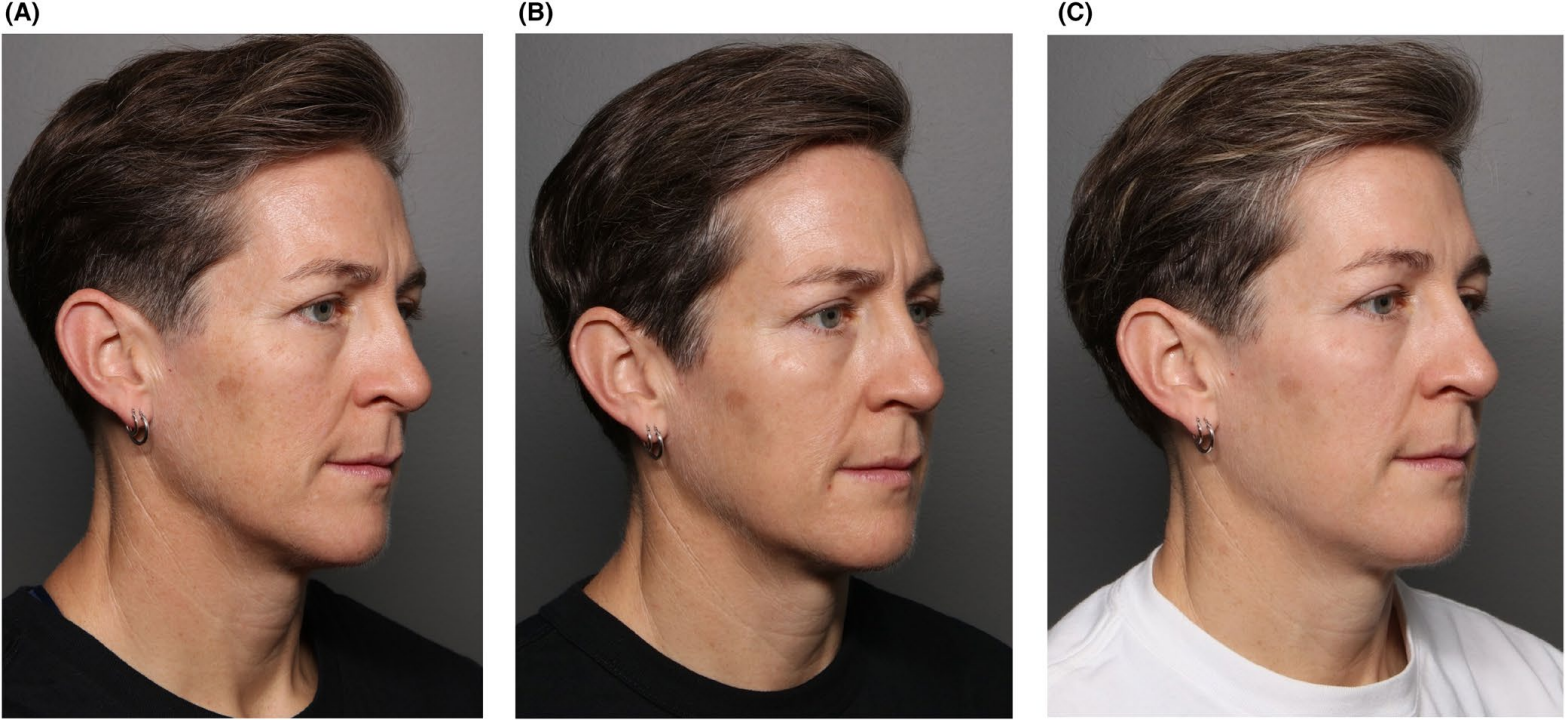
Sequential use of EBD and VYC‐12L as part of the same treatment plan. This 46‐year‐old woman was treated with two different types of EBD (intense pulsed light and fractionated laser; full face), and then 3 weeks later with VYC‐12L (2 mL; cheeks) and onabotulinumtoxinA (42 U; glabella and forehead). The images show her before treatment (A), 3 weeks after treatment with EBD but before the use of injectables (B), and then 4 weeks after treatment with VYC‐12L and onabotulinumtoxinA (C). The impact of adding VYC‐12L is particularly evident in reduced skin dullness (greater radiance), decreased dryness (hydration), and decreased roughness (smoother). Images are courtesy of Shannon Humphrey. EBD, energy‐based device.

**FIGURE 3:**
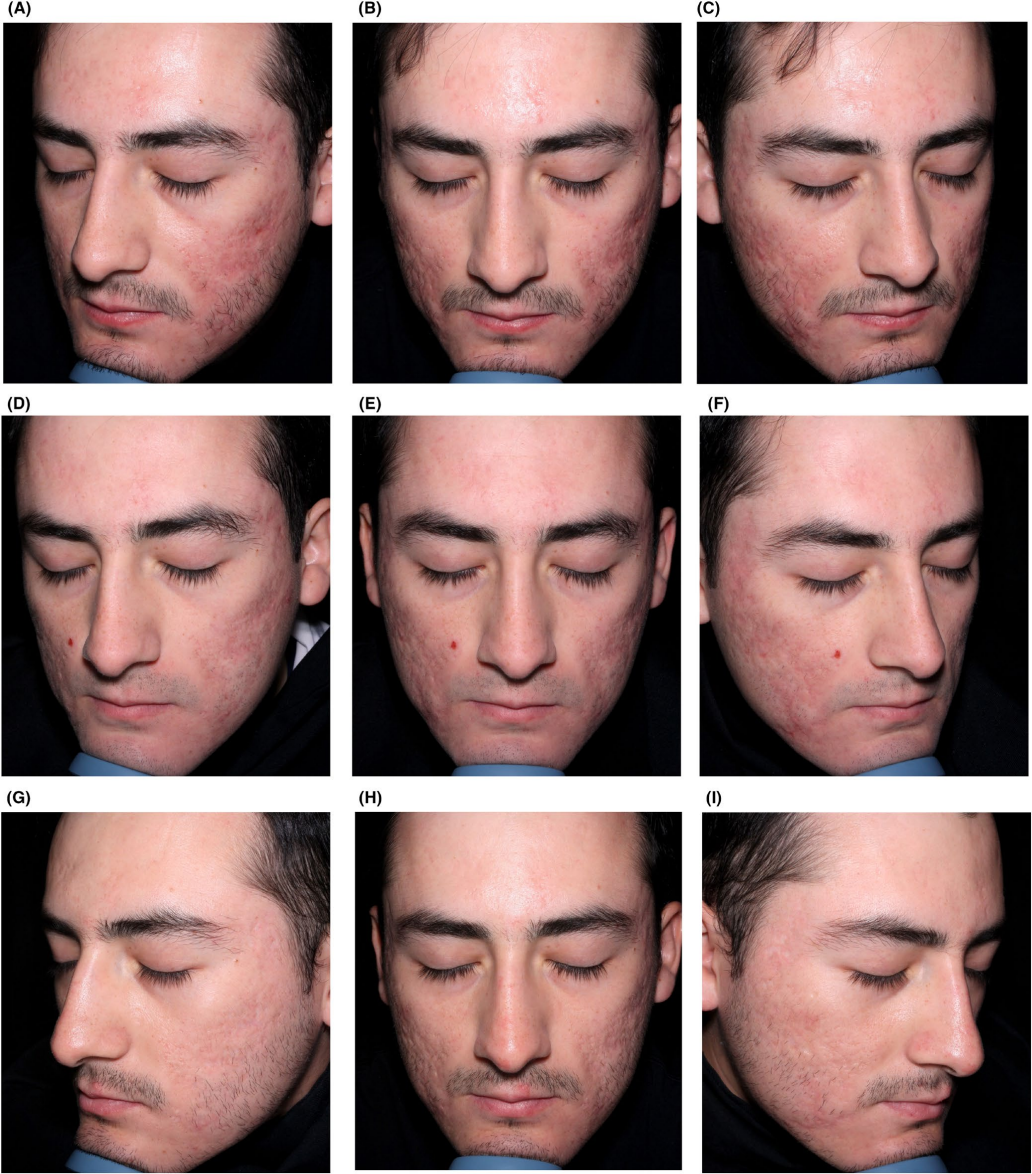
Sequential use of EBD and VYC‐12L as part of the same treatment plan. This 23‐year‐old man was treated with CO_2_ laser (cheeks, temples, nose, chin, and frontal regions), and then 45 days later with VYC‐12L (1 mL per side in the zygomatic malar, submalar, and supramandibular maseteric areas). The images show him before treatment (A–C), 45 days after treatment with CO_2_ laser but before VYC‐12L treatment (D–F), and then 30 days after treatment with VYC‐12L (G–I). In this case, the low risk of causing post‐inflammatory hyperpigmentation with VYC‐12L provided an additional rationale for combining with laser treatment (where the risk may be higher) [5]. The impact of adding VYC‐12L is evident in the improvements to his acne scarring. Images are courtesy of Ileana Arreola Jáuregui. EBD, energy‐based device.

**FIGURE 4:**
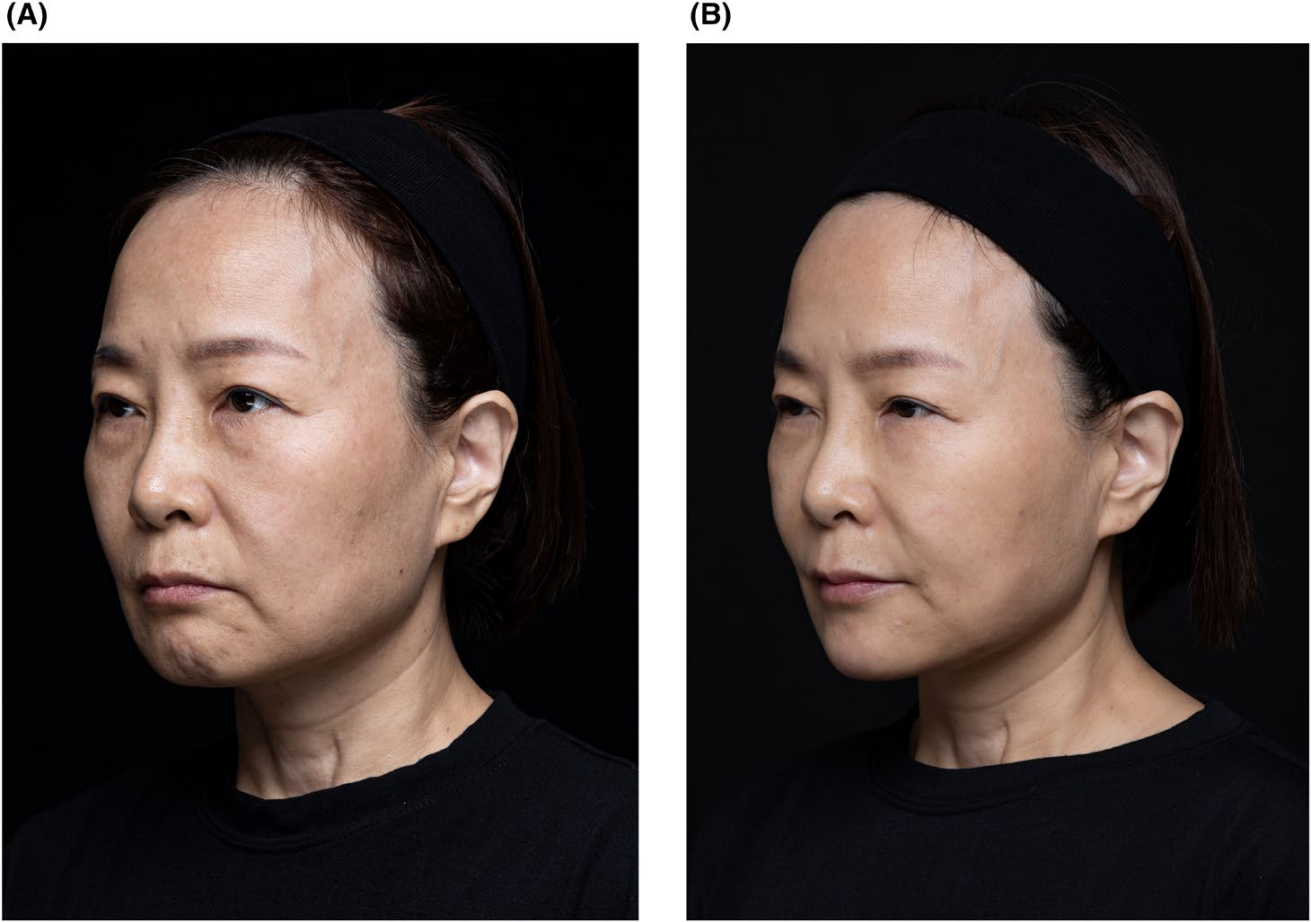
Use of EBD and VYC‐12L within the same treatment session. This 52‐year‐old woman was treated with two different types of EBD (microfocused ultrasound and radiofrequency; full face), Juvéderm fillers (VYC‐20L, 4.5 mL per side in the temples, cheeks, chin, and jaw; VYC‐17.5L, 2 mL per side in the nasolabial folds and lips; VYC‐15L, 0.5 mL per side in the tear troughs), as well as VYC‐12L (cheeks, 2 mL per side), all within the same session. The images show her before treatment (A), and 1‐month post‐treatment (B). The impact of adding VYC‐12L is particularly evident in improved skin texture and reduced roughness. Images are courtesy of Wonseok Choi. EBD, energy‐based device.

Figures 2, 3, 4 show case studies demonstrating the additive benefit of using VYC‐12L and EBDs within the same treatment plan.

Of course, there are also patients in whom co‐treatment is less appropriate—and who may be better managed using either VYC‐12L or EBD alone (Table 4). Key factors to consider include patients' age, phototype, lifestyle, and individual treatment requirements, as well as any financial constraints.

Age is often an important factor. In younger patients with generally good skin, VYC‐12L alone often provides excellent results as a form of ‘prejuvenation’. By contrast, in older individuals, more complex problems are frequently present and EBDs may therefore be necessary. These can be used alone or in conjunction with other treatments as per individual needs. Menopausal estrogen deficiency is often associated with reduced skin barrier function and hence dryness [32], suggesting that VYC‐12L could also be particularly beneficial in these individuals.

In addition, phototype should be considered. Patients with darker skin may be more vulnerable to EBD‐related AEs like hyperpigmentation, particularly with lasers [5]. Where there is specific concern around this risk, VYC‐12L is often the logical “color blind” treatment choice, administered alone or alongside a low‐intensity EBD protocol.

Furthermore, patient availability and desired frequency of visits are relevant to treatment decision‐making. For example, some individuals may only want—or only be available for—a single treatment visit and hence might be particularly suited to VYC‐12L injection (possibly in conjunction with one session of EBD treatment). Others prefer frequent clinic visits, which align well with multisession EBD protocols (with or without VYC‐12L, as appropriate).

Additional patient factors must also be considered, including their desire for rapid results (a relative indication for VYC‐12L), or any significant concerns about bruising or needle‐phobia (relative indications for EBDs).

Budget is often an important issue, and in some cases, it will be necessary to select only the modality likely to give the greatest benefit. VYC‐12L alone may be most appropriate in patients needing treatment for skin dryness, fine lines, or dullness, particularly when they have no other major problems with their skin. By contrast, specific EBDs used alone may be most appropriate for individuals with concerns that are less well treated with VYC‐12L—such as significant laxity or vascular and pigmentation conditions like rosacea, hyperpigmentation, or visible facial veins.

A final—non‐patient‐related—consideration revolves around the capital expenditure of purchasing EBDs. In particular, some new practitioners may be reluctant to make this outlay or be unsure about which device to choose. VYC‐12L therefore provides an excellent alternative for improving skin quality based on a much smaller initial spend.

## 5 Sequencing of VYC‐12L and EBDs Within a Single Treatment Plan

When using VYC‐12L and EBDs in the same treatment plan, it is essential to consider the most appropriate sequencing. However, there is no ‘one size fits all’ approach, and usage should be customized according to the specific EBD(s) being used, as well as patient and practitioner preferences. The injectors in the present author group employ different protocols, and there is a reasonable clinical rationale for performing the VYC‐12L injections: (1) as a standalone at the start, before initiating EBD treatment; (2) on the same day as the first session of EBD; or (3) between EBD sessions or even at the end of the EBD treatment plan. These are briefly considered in turn below.

Two of the current author group sometimes inject VYC‐12L in a separate treatment session before commencing EBD, typically with a time gap of at least 1–2 weeks. The aim here is to leverage the well‐characterized hydrating effects of VYC‐12L [12, 14] before using EBDs, which may be associated with drying of the skin [33]. In addition, HA appears to play an important role in mediating collagen synthesis and structure [28, 30, 34]. Thus, a single session of VYC‐12L treatment may be valuable for dermal “preconditioning”. This has not yet been studied in clinical trials and warrants further investigation.

Six of the eight injectors commonly use VYC‐12L and EBDs on the same day (as long as these are non‐ablative and do not include microneedling). This has the advantage of minimizing the total number of separate treatment sessions and might also maximize short‐term improvement. It should be noted that the VYC‐12L label includes a contraindication for the simultaneous use of lasers [11]. However, a review of the use of HA and laser or IPL on the same day found that while data were limited, they were generally supportive of the safety and effectiveness of this approach [8]. Moreover, same‐day treatments with VYC‐12L (and/or fillers), EBD, and neuromodulators have been shown to confer greater patient satisfaction than sequential usage, with equally predictable outcomes and no unique side effects [20, 31].

When performing same‐day treatments with VYC‐12L and an EBD, most injectors in this group favor the use of the EBD first to ensure precise targeting of the appropriate anatomical layer and a clean field. However, when using epidermis‐targeted IPL or non‐ablative laser therapy, some of the authors prefer the opposite order, based on first injecting VYC‐12L and then applying the EBD. This sequencing facilitates a deep‐to‐superficial treatment order and optimal depth control during VYC‐12L injection; it might also mitigate any possibility of EBD‐related vasodilation leading to increased bruising risk when subsequently injecting VYC‐12L.

Whichever way round the two modalities are used, there should always be a time gap of ≥ 15 min—within the normal cadence of systematic clinical care—to allow sufficient time to cool the skin and re‐prepare it for the second treatment. Indeed, while meticulous, aseptic technique is important when using only one modality, it becomes even more essential when using two or more on the same day.

There remains some debate around using EBDs on skin that has recently been injected with HA. This interaction will likely differ according to the specific EBD and energy level used, the type of HA product and depth of injection, as well as the time gap between each modality [35]. Nonetheless, there are data showing that EBDs can be used immediately after HA injection without compromising the effects of the HA—particularly with superficially targeted laser and IPL treatments [8]. On the flipside, histological analyses have suggested that HA may be damaged by EBD‐induced high temperatures, particularly when the EBD is targeted at deeper tissues [7, 36]. However, these studies were not performed with Vycross fillers. A recent assessment using VYC‐12L found no immediate morphological changes to intradermally administered HA following subsequent treatment with various different EBDs [37]. Furthermore, all Vycross products are heated during product sterilization to > 100°C, so EBDs (which heat the skin to no more than ~65°C) might be expected to impact injected VYC‐12L only minimally. Nonetheless, additional in vivo studies are needed to assess any potential delayed effects of EBDs. Practitioners who are concerned should consider sequencing deep‐targeted EBDs prior to the injection of VYC‐12L.

Indeed, this aligns with the third common pattern of VYC‐12L usage, based on injecting between EBD sessions or at the end of the treatment plan. Such a strategy will be required with ablative lasers or other methods that break the skin (e.g., microneedling with RF), where it is not recommended to inject VYC‐12L on the same day owing to the potential increased risk of AEs. A staggered approach may also be appropriate with patients who are cautious or inexperienced; such individuals often prefer to start with EBD treatment and then decide later whether to add injectables like VYC‐12L, once their confidence has grown. This is particularly common in some countries, for example in parts of Asia, where EBDs are the usual ‘entry point’ for skin‐quality treatments.

In the longer term, periodic treatment reassessment should be built into all patient–practitioner relationships. Depending on the treatment goals, it is often beneficial to repeat multimodal plans based on VYC‐12L and EBDs, with VYC‐12L typically requiring re‐injection every ~6 months. During reassessment, consideration should be given to the success of the original treatment plan; any changes in the patient's needs, lifestyle, or budget; and the EBDs used previously (e.g., it may not be desirable to repeat ablative laser treatment frequently).

## 6 Technical Considerations for VYC‐12L Usage With EBDs

From a technical perspective, the deployment of VYC‐12L in the same treatment plan as EBDs is typically similar to usage alone. The standard protocol is based on a single session of micro‐depot injections into the deep dermis using the 32G ½″ (1.27 cm) needle provided with the product—with entry points spaced 0.5–1.0 cm apart and 0.01–0.05 mL of VYC‐12L deposited in each to ensure uniform distribution [38, 39]. The needle should be inserted at less than 45° to the skin to facilitate the correct (deep dermal) injection depth.

New injectors may be best advised to train and initiate their practice using this pattern. However, there are alternatives, albeit with limited supporting clinical evidence. For example, with regard to the injection device, two of the eight practitioners in the present author group favor the use of a shorter needle (4 mm) in preference to the ½″ (1.27 cm) device; in general, individual injectors are advised to select the needle length that they feel provides the most control, particularly with regard to depth of placement. In addition, six of the current group use a 23–25G cannula for some treatments—particularly in the perioral region, neck, and hands, or for acne scars. Previous guidance recommended against using a cannula, owing to the difficulty of targeting the deep dermis [38], and cannula usage is not currently included in the product labeling [39]. However, many of the present authors note that it is possible to achieve good results through cannula‐based administration into the superficial subdermal tissue (as close as possible to the dermis). In experienced hands, this device may be advantageous in certain circumstances. For example, it reduces the number of entry points, which could be more comfortable for patients, and it typically decreases the risk of bruising and unevenness in individuals (or specific treatment areas) with thinner skin. Furthermore, cannulas have the added benefit of dissecting fibrous areas in patients with acne scarring. On the downside, depth control is more difficult, and more product may be required to get the same results compared with a needle. In addition, there are currently no supporting data, and hence, there is a need for clinical studies assessing the use of cannulas for administering VYC‐12L.

Irrespective of the device used, depth control is absolutely essential to good outcomes. Injecting too deep into the subcutaneous space may reduce effectiveness and increase the likelihood of penetrating a blood vessel, leading to a risk of bleeding and bruising; cases of vascular occlusion are rare [20], but practitioners should be vigilant for warning signs such as blanching [40]. By contrast, injecting too superficially may be associated with product visibility as a bleb or nodule—a phenomenon known as “cobblestoning”. This can often be managed with gentle molding or massaging of the affected area, and if individual blebs persist, it may be possible to treat with hyaluronidase (although this is off‐label in most countries). Such issues are rarely problematic in the hands of the practitioners in the current author group, and should not typically be a major concern with adequate training and optimized technique. Tips for safe and effective use of VYC‐12L are provided in Table 5. These are not mandatory (and are not all applied by each of the present author group), but they may be helpful to individual injectors in their own daily practice. Treatment‐related pain is rarely a significant issue with VYC‐12L, but topical skin‐numbing agents may be applied prior to treatment, if required.

**TABLE 5:** Technical tips with VYC‐12L.

Topical anesthesia may be applied for ~30 min prior to injection, particularly if the patient is concerned about pain Use a grid to standardize the spacing of injection sites, particularly when first using the product Consider possible differences in skin thickness across treatment areas (e.g., typically thinner in the neck), which may affect optimal placement depth Apply gentle pressure to each injection point after withdrawing the needle Mold or massage the treatment area after each injection (particularly if the product has been deposited too superficially) and/or at the end of treatment Pause occasionally during treatment to assess for any developing issues at previous injection sites (e.g., blebs, bleeding, etc.) ○ Consider adjusting injection depth for subsequent injections if needed Change the needle as necessary to ensure it remains sharp (e.g., for every ~1 mL of VYC‐12L used) ○ More frequent changes may be needed with male patients due to their coarser hair follicles and thicker skin Apply a sterile cold compress or ice pack at the end of treatment to calm the skin Have a nurse or assistant on hand during treatment (e.g., to help with pressing out any bleeding points)

The volume of product used should always be tailored according to the treatment surface area, as well as the initial skin quality and patient expectations. In the authors' experience, around 1 mL of VYC‐12L will typically be required for each cheek, 1 mL for the forehead, 1 mL for the perioral area, 1 mL for the periocular area (0.5 mL per side), 1–2 mL for the neck, 2–3 mL for the décolletage, and 1–1.5 mL for each hand. The size of individual micro‐aliquots should be ~0.01 mL [12], but may be customizable up to 0.05 mL. For example, they can be a little larger where there is underlying bone or where the skin is thicker.

Adequate preparation and aseptic technique are essential. When using VYC‐12L and an EBD on the same day, particular consideration should be given to the minimization of contamination risk and to the optimal sequencing of the two modalities (as described in the previous section).

Finally, it is essential to evaluate the results of treatment over time. Patients often make subjective assessments that their skin looks and feels “softer” or “less lined” or “more glowing” in the months after a single session of VYC‐12L injections. It is important to gather such feedback as these are the outcomes that typically matter most to patients; validated tools like SKIN‐Q or the FACE‐Q skin appearance module can support this process [41, 42]. More objective evaluations should also be made, based on standardized before‐and‐after photography [40]. In addition, a number of 2D and 3D imaging systems are available that can provide assessments of some skin‐quality attributes, such as lines and roughness. Furthermore, devices like a corneometer or cutometer may facilitate the assessment of specific attributes [4].

## 7 Patient Education and Advice

It is essential to take a holistic approach to consultation, based on gaining a full understanding of the patient (aesthetic concerns, lifestyle, medical history, treatment goals, etc.), comprehensive assessment of the face and other treatment areas, consideration of all potential treatments, and development of modifiable long‐term treatment plans [43].

Before considering the use of both VYC‐12L and EBDs, any financial or time constraints should be explored. Appropriate education must be provided, covering the mechanisms of action of each modality, as well as the properties of VYC‐12L that make it appropriate for skin‐quality improvement (e.g., relating to elasticity [*G*′], cohesivity, spreadability, and tissue integration [10]). It may also be valuable to explain the key differences between VYC‐12L and other skin treatments marketed for similar purposes—in particular, that the regimen is based on a single session rather than requiring multiple treatment visits 2–4 weeks apart.

The expected benefits of VYC‐12L and the selected EBD(s) should be explained. In particular, it may be worthwhile to cover putative mechanistic synergies (described earlier in this paper), as well as the potential value of treating different quality attributes and at varying anatomical depths.

Treatment plans based on injecting VYC‐12L alongside multiple cycles of an EBD—and potentially also including other aesthetic treatments such as fillers and botulinum neurotoxins—can be quite complex. Hence, patients should be given clear explanations of treatment timings, the downtime (if any) with each modality, the varying timeframes of effect onset, and their likely durations.

It is also important to brief patients on the normal sequelae of each procedure. Individuals who have been adequately prepared for such events may be less disposed to undue concern. Patients must also be educated on the more clinically significant AEs that can occur, although these are typically rare with well‐trained and suitably experienced practitioners [5, 12, 14, 19, 20].

Finally, following each treatment session, all patients should be given sufficient after‐care instructions. Typical guidance with VYC‐12L and non‐ablative EBDs is usually straightforward and may include the avoidance of hot water contact, make‐up, skincare products, strenuous exercise, sun exposure, and excessive touching of treated areas for at least 1–2 days afterwards [38, 40, 44]. With ablative EBD treatments, more intense after‐care will of course be necessary, including detailed guidance on the possible use of cold soaks, ointments, and dressings [45]. Regardless of the modalities employed, appropriate post‐treatment follow‐up plans should always be in place.

Among patients who have been appropriately selected and properly managed, the injectors in the author group have found that satisfaction rates with VYC‐12L and EBD are typically high. Many individuals subsequently pinpoint VYC‐12L as the modality that made the most difference to their skin, and specifically request it again when they return in the following months.

## 8 Limitations and Future Work

The present paper synthesizes the extensive knowledge and experience with VYC‐12L and different types of EBD among a global group of practitioners. Nonetheless, there remain limited data from clinical studies on the use of VYC‐12L and EBDs within the same treatment plan. Although several real‐world analyses have been published [20, 21, 22], no prospective, controlled trials have yet been performed. Such studies would be highly valuable. In their absence, the authors encourage continued collation of results from routine practice, examining the effectiveness, patient‐reported outcomes (including satisfaction with results), and safety of integrating these modalities. In particular, there is a need to compare outcomes with EBDs and VYC‐12L deployed within the same treatment plan versus single modalities used alone. It will also be important to evaluate the relative utility of different sequences and protocols, and to assess outcomes with cannula‐based administration of VYC‐12L.

## 9 Conclusions

**FIGURE 5:**
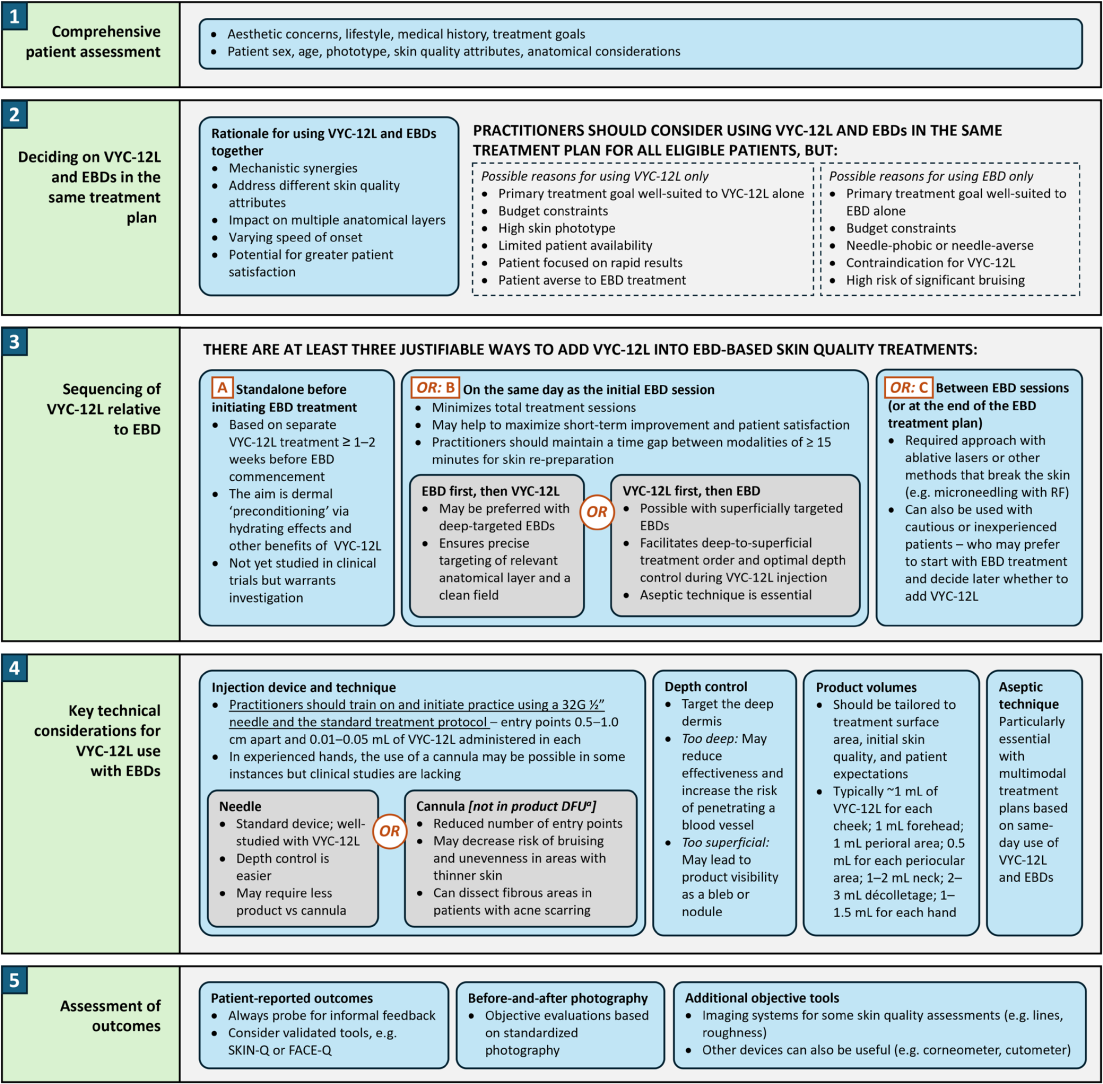
Summary: Five essentials when using VYC‐12L and EBDs in the same treatment plan. ^a^Cannula‐based administration of VYC‐12L has not been assessed in clinical trials and is not currently included in the product labeling [39]. DFU, directions for use; EBD, energy‐based device; RF, radiofrequency.

There is a strong rationale for using VYC‐12L and EBDs in the same treatment plan for skin‐quality improvement. This is based on potential mechanistic synergies (particularly relating to the hygroscopic effects of HA and increased expression of AQP3 with VYC‐12L), as well as complementary abilities to address different attributes and multiple anatomical layers (Figure 5). Careful consideration should always be given to appropriate sequencing. VYC‐12L and non‐ablative EBDs are often used on the same day, but treatment fields must be appropriately managed and principles of aseptic technique meticulously observed. VYC‐12L is associated with low rates of AEs, but maintaining the correct injection depth is central to optimal outcomes; practitioners should ensure that they receive adequate training and select the injection tools that give them the best control. Overall, multimodal treatment using VYC‐12L and EBDs together can provide a more comprehensive approach, with high levels of resulting patient satisfaction.

## Author Contributions

All authors contributed to the conception and design of the paper and the interpretations provided; were involved in drafting the manuscript and revising it critically for important intellectual content; gave final approval of the version to be published; and agreed to be accountable for all aspects of the work.

## Ethics Statement

The authors have nothing to report.

## Consent

All of the patients whose photographs are used in this publication provided written informed consent.

## Conflicts of Interest

Shannon Humphrey is a consultant, speaker, and investigator for Allergan Aesthetics, an AbbVie Company. Sylvia Ramirez is a consultant, speaker, and investigator for Allergan Aesthetics, an AbbVie company. Ileana Arreola Jáuregui is a speaker for AbbVie, Galderma, Pierre Fabre, Leo Pharma, ISDIN, Eucerin, and Panalab. Wonseok Choi reports nothing to disclose. Krishan M. Kapoor is a trainer and speaker for Allergan Aesthetics, an AbbVie company. Mansi Mukherjee is a consultant and speaker for Allergan Aesthetics, an AbbVie company. Fabiana Wanick is a consultant, speaker, and investigator for Allergan Aesthetics, an AbbVie Company. Reha Yavuzer is a consultant, speaker, and investigator for Allergan Aesthetics, an AbbVie Company. Smita Chawla and Carola de la Guardia are employees of Allergan Aesthetics, an AbbVie company, and own AbbVie stock.

## Data Availability

Data sharing is not applicable as no new data were generated. AbbVie is committed to responsible data sharing regarding the clinical trials we sponsor. This includes access to anonymized, individual, and trial‐level data (analysis data sets), as well as other information (e.g., protocols, clinical study reports, or analysis plans), as long as the trials are not part of an ongoing or planned regulatory submission. This includes requests for clinical trial data for unlicensed products and indications. These clinical trial data can be requested by any qualified researchers who engage in rigorous, independent, scientific research, and will be provided following review and approval of a research proposal, Statistical Analysis Plan (SAP), and execution of a Data Sharing Agreement (DSA). Data requests can be submitted at any time after approval in the US and Europe and after acceptance of this manuscript for publication. The data will be accessible for 12 months, with possible extensions considered. For more information on the process or to submit a request, visit the following link: https://vivli.org/ourmember/abbvie/ then select “Home”.
